# Analysing loneliness forum posts, the comments they elicit, and the responses to these comments

**DOI:** 10.1371/journal.pmen.0000037

**Published:** 2024-11-06

**Authors:** Praise-EL Michaels, Natasha Tonge, Oluwadara Kalejaiye, Aaryan Panthi, Rahual Rai, Mmekut-Mfon Gabriel Edet, Erika Ferguson, Legand Burge, Gloria Washington, Anietie Andy

**Affiliations:** 1 Electrical Engineering and Computer Science, Howard University, Washington, District of Columbia, United States of America; 2 Clinical Psychology, George Mason University, Fairfax, Virginia, United States of America; 3 Moniepoint, Lagos, Nigeria; University of Canterbury, NEW ZEALAND

## Abstract

In May 2023, the United States surgeon general put out an advisory suggesting that loneliness and social isolation should be tackled and prioritized like health conditions such as substance abuse. Online loneliness forums provide a platform for users experiencing loneliness to engage with, share experiences/concerns, and seek support from others. On these forums, users (posters) publish support seeking posts and other users respond by either reacting to these posts and/or writing comments in which they provide support. In some cases, the posters respond to and engage with the comments that their posts elicit; understanding these interactions between posters and the comments their posts elicit can inform helpful communication strategies on online loneliness forums and online health forums, in general. Prior work on analyzing data from online loneliness forums did not study the interactions between posters and the comments their posts elicit. To address this, we present a dataset of posts and comments published in a 4 year time period i.e. from January 1 2019 to December 31 2022 on an online loneliness forum on Reddit. This dataset consists of (a) posts, (b) all the comments associated with these posts, (c) the comments that elicited responses from the posters, and (d) the responses of the posters to these comments. With this dataset, we conduct analysis using a topic modeling algorithm called BERTopic and a psycholinguistic dictionary called Linguistic Inquiry and word count (LIWC) to gain insights and elucidate the language markers associated with comments (to posts) that elicit responses from the posters. We find that as it relates to comments that received responses from posters, the following topic themes were associated with these comments i.e. relationships, empathy, and mental health concerns and the LIWC categories on second person pronoun, social processes, and present focus, were associated with these comments. We also find that the topic themes on appreciation was associated with the responses by posters to comments their posts received. We discuss these findings in the discussion section.

## Introduction

Loneliness, which is defined as the discrepancy between a person’s desired and actual social relationships has been linked to early death [1], depression [2], and heart disease [3]. Prior works have shown an increase in the expression of loneliness by individuals since the beginning of the COVID-19 pandemic [4–8].

Some individuals who experience loneliness seek support on online loneliness forums such as */r/Lonely* on Reddit—which is the loneliness-related forum on Reddit with the most number subscribers i.e. over 370,000, as of February 2024. These online loneliness forums serve as a sanctuary for individuals—some of whom are from various geographical locations, to interact with others, share/exchange their experiences, and seek or provide support around loneliness. Several prior works have studied how users communicate—as it relates to loneliness, on social media platforms and online loneliness forums [9–16]; these prior works will be discussed in the related works section. There are several benefits to understanding how users communicate around loneliness on social media and on online forums. For example: (a) in [13], it was determined that individuals who seek support on Twitter around loneliness and belong to different age groups, and genders, express and seek different kinds of support and (b) in [10], individuals who seek support on online loneliness forums also seek loneliness-related support on online general forums. Given the findings from these studies (i.e. (a) and (b)), online loneliness interventions should be designed in such a way that the interventions vary by age group; also, posts published on online general forums by individuals who express the feeling of loneliness on online loneliness forums should be studied to gain a better understanding of the kinds of support these individuals need. Prior work did not study the interactions between posters and the comments their posts receive. On online loneliness forums, users (posters) publish support seeking posts and other users provide support by either reacting to and/or responding to these posts with comments; this work focuses on the comment responses to posts. Sometimes, the posters respond to and engage with the comments associated with their posts. Tables 1 and 2 show examples of interactions in which a poster responds to a comment their post received.

**Table 1:** Example 1 of a poster responding to a comment their post received.

User	Post/Comment/Response	Text
User A	Post	“*Today’s my (f) 34th birthday. I have no real friends, I’m single, and not anywhere near where I thought I’d be at this age. This just sucks and hurts a lot. Sorry for the little vent, no one to talk to*”
User Z	Comment	“*Happy Birthday!* ☺ *It was my birthday last Friday and I spent it just working and reading books. No friends visited me, nobody gave me anything more than a text or maybe a 10 second call*”
User A	Response	“*Thank you, and happy late birthday! I’m sorry about your friends*”

**Table 2:** Example 2 of a poster responding to a comment their post received.

User	Post/Comment/Response	Text
User B	Post	“*I just left my only “friend” after refusing to be treated like shit all the time and ignored, if you’re wondering why I still stuck around it’s because I had literally no one else. Now I’m left with no one. It was really hard and actually my hands are still shaking after typing the last message to her. So I’d appreciate if y’all could say something, whatever it is …*”
User Y	Comment	“*Having no one is better than having toxic friends*”
User B	Response	“*Definitely but everytime I get hope that maybe I can make some real genuine friends they always leave me or end up being toxic so I have to leave them instead. They always appear sweet and caring at first but then reveal their true colors when they get bored of me or some shit, I don’t understand at all!*”

There are benefits to understanding the engagement/interactions between posters and the comments their posts elicit on online loneliness forums. For example, understanding these interactions can (a) inform helpful communication strategies on online loneliness forums and online health forums, in general and (b) inform the design and implementation of online loneliness interventions. A recent meta-analysis found that of the many effective strategies for reducing loneliness, online strategies have so far been ineffective [17]. Poor understanding of the dynamics of online communities sought out by people who are lonely may be impeding the development of effective online interventions. By creating and analysing a dataset of the interactions between posters, their posts, and the comments their posts receive, we aim to bridge this gap. In the current study we use BERTopic [18] and LIWC [19], a psycholinguistic dictionary consisting of more than 70 categories e.g. health, and a collection of words associated with each of these categories to better understand the dynamics of engagement in the loneliness forum.

## Ethics

The Institutional Review Board guidelines of the authors institutions considered this work to be exempt. The data used in this work are publicly available. No user or moderator in the */r/Lonely* forum were contacted before, during, and after this study. Given that the content of the posts, comments, and responses to the comments were sometimes sensitive, we met weekly with the team working on this project to discuss and ensure that they were not negatively affected by the content of the data used in this work.

## Related work

Prior work has been done to gain insights into how individuals communicate around loneliness on online loneliness forums and on social media platforms. In [13], it was determined that individuals who express loneliness on Twitter and belong to different age groups and genders seek different kinds of support as it relates to loneliness. In [11], Twitter posts of individuals who conveyed the feeling of loneliness were analyzed and it was determined that there was a correlation between their posts and predictors of mental health: Loneliness is comorbid with depression. In [10], online loneliness forum posts by users who self-declared to experience loneliness and depression where compared to online loneliness forum posts by users who self-declared to experience loneliness but not depression. It was determined that users belonging to these groups seek different kinds of support. Some individuals who seek loneliness-related support on online loneliness forums also seek loneliness related support on non-loneliness online forums [9]. In [12], Facebook posts of individuals who experience loneliness were analyzed and it was determined that the language used in expressions of loneliness was similar to that used in the expression of depression; however, the language used in expressions of loneliness tended to focus more on cognitive process compared to the language used in expressions of depression.

[20] highlights the increasing use of the internet by older adults, 50 years and older, with significant portions using it for health-related information and emotional support. The study used qualitative content analysis to analyze 400 messages from the SeniorNet discussion board about depression. An inductive approach was used to develop a code scheme, and inter-coder reliability was tested to ensure consistency. By investigating the components and patterns of empathy in online communication, this study sheds light on the complex dynamics of online communities and provides valuable insights for improving social interaction and emotional support in digital environments. In [21], they sought to explore the process by which individuals in an online support community transition from being receivers of support to becoming givers, with a specific focus on the concept of reciprocity. The study examines how social support and reciprocity can reduce feelings of loneliness and improve mental well-being in individuals who are part of an online support community. It examines how receiving and providing support impacts members’ sense of belonging and psychological health. It also highlights the importance of social attachment and shared experiences in fostering sustained participation and contribution. The results have practical implications for the creation of online communities and theoretical implications for comprehending the dynamics of reciprocity and role transitions. In [22], hypotheses were presented on the impacts of relationship characteristics (relationship orientation, self-disclosure, and networking ability) and online social network (OSN) features (active and passive features) on social loneliness. It is also said that people have different ideas about how much reciprocity is needed. This creates two types of relationship orientations: communal and exchange. A communal orientation means that the person usually does not need immediate reciprocation for a benefit they provide but still looks out for other people’s welfare. An exchange orientation, on the other hand, means that the person maintains relationships based on reciprocation and instrumental reasons. This led to the formulation of several hypotheses under the three relationship characteristics previously named above, with the most relevant hypotheses for us in this context being the two hypotheses that fall under the moderating factor of loneliness (self-disclosure). They are: (a.) Self-disclosure moderates the relationship between communal relationship orientation and perceived social loneliness, such that the relationship becomes stronger when there are higher levels of self-disclosure. (b.) Self-disclosure moderates the relationship between exchange relationship orientation and perceived social loneliness, such that the relationship becomes stronger when there are higher levels of self-disclosure.

These prior works did not study the interaction between posters and the comments their posts elicit. This work is different from prior work in that the objective of this work is to examine the interaction between posters and the comments their posts elicit.

## Dataset

We collected posts and comments (including metadata associated with these posts and comments e.g. username) that were published from January 1 2019 to December 31 2022. This dataset is publicly available and the collection and analysis method complied with the terms and conditions for the source of the data. These posts and comments were written in English. Table 3 shows the total number of posts we collected and the number of users who published these posts. Table 4 shows the number of comments these posts received and the number of users who published these comments.

**Table 3:** Total number of posts collected on the Reddit Loneliness forum, /r/Lonely from January 2019 to December 2022.

2019–2022	No_of_Posts	No_Users
Total	209,430	64,324

**Table 4:** Total number of comments on the Reddit Loneliness forum, /r/Lonely from January 2019 to December 2022.

2019–2022	No_of_Comments	No_Users
Total	1,338,331	164,800

The aim of this work is to gain insights about the interactions between posters and the comments their post receive. Hence, in order to obtain the data for this analysis, we identified posts in which the poster responded to at least one of the comments their post received. To do this, we did the following:

1. From the posts (Table 3) and comments (Table 4), we varied the number of comments posts received—starting at 5 with increments of 5 and observed that posters who published posts that received 30 or more comments were more likely to respond to at least one of the comments. Hence, we identified and collected 6,068 posts (i.e. 2.89% of the total number of posts collected) that received 30 or more comments (i.e. 438,811 comments or 32.79% of the total comments collected).
2. From the dataset in (1) above, we identify the posts where the poster responded to at least one comment; we did this by (a) writing code to identify the posts in which a poster responded to at least one comment and (b) we split these posts and comments into three (sets) and had 3 student volunteers review each set and confirm that posters responded to at least one of the comments. When a student was done reviewing a set of posts, we had the other two (2) students review the students’ work to resolve any issues. If a discrepancy was observed, the posts and comments with the discrepancy were discussed by all the students and a resolution was made based on the discussions.

Table 5 shows the number of posts published from January 2019 to December 2022 that received 30 or more comments in which the posters responded to at least one of the comments their posts received and Table 6 shows the number of comments these posts received. We use the data from Tables 5 and 6 for the analyses in this work.

**Table 5:** Total number of posts (that received 30 or more comments) published from January 2019 to December 2022 in which the posters responded to at least one of the comments their posts received.

2019–2022	No_of_Posts	No_Users
Total	2,261	1,999

**Table 6:** Total number of comments received from January 2019 to December 2022 by posts in which the poster responded to at least one of the comments.

2019–2022	No_of_Comments	No_Users
Total	27,683	12,257

## Analysis

We conducted two sets of analyses using the topic modeling algorithm, BERTopic [18] and LIWC [19]. We analyzed (a) the comments (to posts) that elicited responses from the posters and (b) the response by posters to these comments. Because loneliness is highly comorbid with depression, we conducted additional analyses to determine if/how users who express loneliness and depression respond to support—provided via comments, to their posts compared to users who express loneliness but not depression. Before we carried out these analysis, we randomly selected 200 posts and the comments that they received. One of the co-authors reviewed these posts and the comments they received and observed that the comments that received responses from the poster and those that did not receive responses from the poster addressed the need expressed in the post.

The data collection and all the analyses conducted in this work complied with the terms and conditions for the source of the data.

### Topic modeling

Using the algorithm, BERTopic [18], which is a topic modeling approach that leverages transformer-based embeddings to generate interpretable topics and topic distributions, we conduct analysis to: (a) determine the topics most associated with comments that elicit responses from the posters and (b) determine the topics most associated with the responses (by posters) to comments their posts received. Specifically, we do the following to apply BERTopic: we first used the Sentence-Bert model to generate document embeddings, we then used Uniform Manifold Approximation and Projection (UMAP) to reduce the dimensionality of the embeddings. The HDBSCAN clustering algorithm was used to cluster the reduced embeddings. Topics were then extracted using the class-based Term Frequency-Inverse Document Frequency (c-TF-IDF). We had two experts with experience in Public Health and with graduate degrees manually review and validate the topics and associated words.

#### Topics associated with comments that elicit responses from posters

Here, we apply BERTopic to (a) the comments (to posts) that received responses from the posters and (b) the comments (to posts) that did not receive responses from the posters. Table 7 shows the top topics associated with comments that received responses from posters; Table 7 only displays the topics associated with comments that received responses from posters and that were not associated with comments that did not receive responses from the posters.

**Table 7:** Top topics most associated with comments that received responses from posters.

Topic #	Topic words
1	friends, friend, friendship, friendships, had, they, them, any, new, have
2	relationship, girlfriend, young, gf, age, never, had, 16, 19, love
3	play, games, game, switch, minecraft, playing, video, apex, legends, console
4	they, hate, them, care, people, empathy, treated, others, asshole, nice
5	men, women, woman, gender, female, society, we, male, man, our
6	hope, day, birthday, great, happy, amazing, good, enjoy, have, fantastic
7	apps, dating, app, using, matches, men, on, use, women, online
8	peace, change, positive, life, solution, mindset, changes, better, negative, thing
9	therapist, therapy, therapists, help, health, mental, psychololgist, trauma, tried, cbt
10	dad, parents, family, kids, sister, your, mom, siblings, sibling, vacation

#### Topics associated with poster responses to comments their posts received

In this section, we apply BERTopic to the responses—by posters, to comments that their posts received. Table 8 shows the top topics associated with poster responses to the comments their posts receive. The findings from this analysis are discussed in the discussion section.

**Table 8:** Top topics most associated with responses by posters to comments.

Topic #	Topic words
1	brother, sister, thanx, thanks, thankyouuu, brotheri, botswana, thankyouuuuu, bra
2	birthday, happy, birthdays, belated, year, wish, celebrate, hope, day, wishes
3	conversation, talk, they, talking, them, conversations, ask, text, call, texting
4	hugs, hug, sending, virtual, back, aww, hugged, big, lovely, huggies
5	thank, you, luv, commenter, track, glad, thanks, right, well
6	support, words, kind, appreciate, thank, glad, much, grateful, your, community
7	loneliness, lonely, felt, eachother, being, other, feel, feeling, sub, arent
8	live, country, UK, from, America, India, Europe, Canada, visit, where
9	advice, tip, thanks, tips, ill, mind, appreciate, take, noted, thank
10	therapy, therapist, therapists, bipolar, bpd, mental, psych, professional, treatment, meds

### LIWC analysis

In this section, similar to prior work [10–12], we use LIWC [19] to determine the LIWC categories most associated with comments that elicit responses from the posters compared to the comments that do not elicit responses from posters, as shown in Table 9. This was done by determining the proportion of words that are associated with the LIWC categories in the comments that elicit responses from posters compared to those associated with comments that did not elicit responses from posters.

**Table 9:** LIWC categories most associated with comments that received responses from posterss.

LIWC Categories	Cohen’s D
Second Person Pronoun	0.180
Function	0.125
Common Verbs	0.111
Social Processes	0.109
Present Focus	0.100

In this paper, we only show the LIWC categories associated with comments that elicit responses from posters. The effect sizes were measured using Cohen’s D, which indicates the standardized difference between two means, and only results with Cohen’s D above or equal to 0.100 are reported. To indicate meaningful correlations, Benjamini-Hochberg p-correction (which is a statistical method that is used to decrease the false discovery rate) was used with p<0.001 as our significance level.

### Loneliness and depression analysis

Given the comorbidity between loneliness and depression, the goal in this section is to gain insights into how users who express loneliness and depression interact with comment responses to their posts in the online loneliness forum compared to users who experience loneliness but not depression. In order to do this, we first identify users who express loneliness and depression and users who express loneliness but not depression. Similar to prior work [10] we make the following assumption: users who publish posts (at the same time period) in a loneliness forum and a forum focused on discussions around depression experience both loneliness and depression. However, users who publish posts in a loneliness forum but do not have any published posts in a depression forum, experience loneliness but not depression. From Table 5, which shows the number of posters and the number of posts with 30 or more comments in which the posters responded to at least one of the comments, we identified the users who published in the loneliness forum */r/Lonely* and a depression forum on Reddit called */r/depression*. We chose the depression forum, */r/depression* because prior work [10] determined that this is the Reddit depression forum most used by users who publish posts in the Reddit forum */r/Lonely*. We observed that of the 1,999 users who published posts in the loneliness forum, */r/Lonely* and responded to at least one of the comments i.e. Table 5, 77 of these users also published posts in the Reddit */r/depression* forum around the same time period i.e. from January 2019 to December 2022. This suggests that users who express loneliness and not depression interact more with the comments their posts elicit compared to users who experience loneliness and depression.

## Discussion, limitations, and future work

In this work, we use language features to understand the interaction between posters, the comments their posts receive, and their responses to these comments. We find that the topics themes most associated with comments that received responses from posters i.e. Table 7 were on friendship, relationships, empathy and how people are treated, dating, therapists and mental health concerns, and family, for example. These findings indicate that posters tended to respond and engage with comments to their posts related to these topics. The finding on posters responding to comments that make reference to mental health concerns indicates that on this loneliness online forum, users seek and respond to support around mental health concerns. Hence, when designing and implementing interventions around loneliness, it is important to know that posters seek support and respond to mental health related concerns.

The responses by posters to comments were analyzed and the topic themes most associated with the responses from posters were on: showing appreciation, talking/conversation, mentions of various countries, and mental health, for example as shown in Table 8. The topic on mentions of various countries indicate that the posters seeking support on the */r/Lonely* forum reside in/are from various countries.

We conducted an analysis to determine the LIWC categories most associated with comments that received responses from posters compared to comments that did not receive responses. We find that the LIWC categories on second person pronoun, function, common verbs, social processes, and present focus were more associated with comments that received responses from posters compared to the comments that did not receive responses from posters. The findings on the LIWC categories on Social Processes and Present Focus being associated with comments that receive responses from posters aligns with prior works which indicated that users who express loneliness on an online loneliness forum tend to use words associated with the LIWC categories on Social Processes and Present Focus [13]. Despite the high comorbidity of loneliness and depression, this finding is notably *in contrast* to prior findings that people who are depressed engage in more first-person pronoun usage [23]. This suggests notable differences between the response behavior of those who are lonely from those who also suffer from depression.

Additionally, we observed that users who experience loneliness and not depression engage more with the comments that their posts received in the loneliness forum compared to users who experienced loneliness and depression. Taken together, the results suggest more active social engagement behavior from lonely compared to lonely and depressed people in online communities. Our results also align with research on social engagement and loneliness. Loneliness may increase attention to social cues [24] and drive a desire to reconnect with others. However, some people do not increase social engagement when lonely, as expected. Previous research has described two general styles of social engagement patterns used by people who are lonely: a prevention-focused style characterized by social avoidance and a promotion-focused style characterized by increasing social engagement when lonely [25]. Some of these studies do not account for depression mixed with loneliness, however [25]. Our results may be reflecting this difference between loneliness and depression mixed with loneliness linguistically: people who are lonely but not depressed demonstrate a promotion-focused style of increased social engagement and this results in more verb use and second person pronoun use. By contrast, people with a prevention-focused style might be more depressed and more self-focused. Our results also show the impact of these styles—the more successful lonely people engage others more with active language that isn’t overly self-focused.

In this study, we employed machine learning techniques to delve into the intricate dynamics of user engagement in a Reddit Loneliness forum, specifically focusing on understanding why certain comments receive responses from the original posters. The outcomes shed light on several noteworthy aspects, but it’s crucial to consider the study’s limitations and potential avenues for future research.

Some of the limitations of this work are:

- The analysis relies on data from a particular Reddit Loneliness forum. Generalizing the findings to other forums or online platforms requires caution, as user behaviors and interactions may vary across forums.
- The study is confined to a specific timeframe i.e. 2019 to 2022. Emerging trends or changes in forum culture, are not explicitly addressed and may influence user behaviors.
- Our analysis is on users with posts that received 30 or more comments and so we cannot extrapolate on posters (with less than 30 comments) that responded to at least one of the comments.
- The individuals who express loneliness and seek support on */r/Lonely* are not representative of the population of individuals who express loneliness. Hence, the findings from this work may not apply to individuals who express and seek support around loneliness but not on online forums or social media.
- The distribution of user contributions per comment in this study was not examined.

In the future, we will include multiple online loneliness forums in our analysis—which will provide a broader perspective on user behaviors, thereby allowing for more comprehensive understanding of the factors influencing engagement on these forums. Individuals belonging to different age groups and genders seek different kinds of support as it relates to loneliness [13]. In the future, we will study how race/ethnicity, age, and gender may influence how posters respond to comments their posts elicit.

## Conclusion

In conclusion, this work studied the engagement between posters, the comments their posts receive, and the posters responses to these comments. The findings from this work can aid in understanding how posters engage with the support they receive via comments and help guide and inform communication strategies on online loneliness forums.

## Supporting information

S1 File Final posts. (XLSX)

S2 File Comments with responses. (CSV)
